# American Canine Hepatozoonosis Causes Multifocal Periosteal Proliferation on CT: A Case Report of 4 Dogs

**DOI:** 10.3389/fvets.2022.872778

**Published:** 2022-04-27

**Authors:** Cambridge L. Coy, Jeremy B. Evans, Alison M. Lee, Danielle R. Dugat, Jonathan M. Levine, John F. Griffin

**Affiliations:** ^1^Department of Small Animal Clinical Sciences, Texas A&M University, College Station, TX, United States; ^2^Department of Clinical Sciences, Mississippi State University, Starkville, MS, United States; ^3^Department of Veterinary Clinical Sciences, Center for Veterinary Health Sciences, Oklahoma State University, Stillwater, OK, United States; ^4^Department of Large Animal Clinical Sciences, Texas A&M University, College Station, TX, United States

**Keywords:** pseudocortex, periosteum, neutrophilia, canis, gulf coast, tick, case report

## Abstract

American canine hepatozoonosis (ACH) represents an important but relatively uncommon differential diagnosis in a dog with fever, muscle wasting, profound leukocytosis, and/or musculoskeletal pain. Despite this, obtaining a definitive diagnosis can prove difficult. Peripheral blood smears and whole-blood polymerase chain reaction (PCR) rely on rare parasitemia, and the gold standard diagnostic test (skeletal muscle biopsy) is uncommonly pursued due to its invasive and costly nature. Demonstration of characteristic periosteal proliferative lesions aids diagnosis. The lesions typically involve the more proximal long bones of the appendicular skeleton. The periosteal proliferation is of currently unknown pathogenesis, but its distribution is characteristic of this disease with few differential diagnoses. This case series describes the findings on computed tomography (CT) in 4 dogs with PCR- or cytologically-confirmed *Hepatozoon americanum*. All dogs had multifocal, bilaterally asymmetric, irregularly marginated, non-destructive, non-articular, periosteal proliferative lesions. Recognition of this unusual CT finding and awareness of this disease could assist in the diagnosis and subsequent treatment of dogs with ACH and may offer an additional indication for CT in cases of fever, muscle wasting, and myalgia.

## Introduction

American canine hepatozoonosis (ACH) is a vector-borne disease caused by *Hepatozoon americanum*, an apicomplexan protozoan from the family Hepatozoidae (1, 2). The first case was reported in 1978 in Texas and was mistaken for *Hepatozoon canis*. Cases of ACH typically occur within the geographic range of the Gulf Coast tick (*Amblyomma maculatum*), which extends along the Gulf of Mexico and southern half of the Atlantic Coast of the United States and also includes all of Arkansas and most of Oklahoma (3). Following ingestion of the Gulf Coast tick, *H. americanum* oocysts rupture and release sporocysts containing sporozoites (4). Sporozoites migrate from the intestines to skeletal muscle and undergo merogony, forming classic “onion skin” cysts identified as early as 3 weeks post-infection (4, 5). In contrast to the mild clinical signs seen with *H. canis* infection, *H. americanum* is associated with severe debilitating systemic illness and is generally fatal. It is rarely cured, and therapy consists of weeks of ponazuril or “triple therapy” (trimethoprim-sulfadiazine (TMS), clindamycin, and pyrimethamine) followed by long-term decoquinate administration (6). Dogs with ACH can have lameness, myositis, and muscle atrophy and may present with myalgia, intermittent fever, and severe mature neutrophilia. An unusual finding of ACH is periosteal proliferation, often identified on radiographs affecting the long bones (7). Clinical signs and periosteal proliferation can develop as early as 4–5 weeks post-infection (5).

Diagnosis can be achieved by microscopic visualization of gamonts within leukocytes on a peripheral blood smear, however, this is low-yield with <0.1% of leukocytes containing intracellular gamonts (8). Whole-blood polymerase chain reaction (PCR) and serologic detection of antibodies have been used, but the sensitivity and specificity of PCR are unknown and immunoassays are not commercially available. Currently, identification of cysts on muscle biopsy remains the gold standard (9). Radiographic detection of the characteristic periosteal proliferation in a dog with a profound peripheral neutrophilia is often sufficient for a presumptive diagnosis of ACH (4). In a retrospective case series (in which dogs were described to be infected with *H. canis* but in hindsight likely had ACH), standard radiographs identified this finding in ~80% (18/22) of dogs (10). In addition to radiographs, nuclear scintigraphy has also been used to characterize bone lesions in dogs with ACH (11).

Computed tomography (CT) has an overall greater sensitivity than radiography for spinal cord diseases and may be preferred over magnetic resonance imaging in cases of subtle bone lesions, such as vertebral subluxations or fractures (12). As computed tomography becomes more commonly utilized for complex cases of spinal pain, fever, and lameness, and dogs may have an unknown travel history or place of origin, it is beneficial to report CT findings in dogs with ACH. This is the first time CT changes associated with ACH have been described in the literature.

## Case Descriptions and Diagnostic Assessment

### Dog 1

A 2-year-old female spayed miniature Australian shepherd dog weighing 9.3 kg presented for a 7-month history of cyclic fever, paresis, and a stiff gait. The dog lived in western Louisiana. The dog was known to be positive for the multidrug resistance mutation (MDR1). Prior serial clinicopathologic abnormalities included severe mature neutrophilia, normocytic normochromic non-regenerative anemia, mild elevation of alkaline phosphatase, mild hyperglobulinemia, and hypercholesterolemia. Creatinine phosphokinase was within normal limits (106 IU/L [10–200]). Laboratory values for all cases are summarized in Table 1. Thoracic radiographs were unremarkable with the exception of spondylosis deformans of the C6–C7 intervertebral disc space. Radiographs and arthrocentesis of stifles and carpi were unremarkable. Abdominal ultrasound noted suspected microhepatia and a small left adrenal gland nodule (thought to be incidental). Urinalysis, quantified urine culture, serum cortisol, and serum total T4 were all within normal limits. An atlanto-occipital cerebrospinal fluid sample was unremarkable. Despite numerous courses of empiric antimicrobial therapy (including amoxicillin/clavulanate, enrofloxacin, cefovecin sodium, doxycycline, and ketoconazole), clinical signs persisted. There was transient improvement noted during a previous course of prednisone. Upon arrival, the dog was receiving ~0.5 mg/kg/day of prednisone.

**Table 1:** Comparison of pertinent laboratory values.

	**Dog 1**	**Dog 2**	**Dog 3**	**Dog 4**
Packed cell volume (%)	31	41	38.9	42
Plasma protein (g/dl)	7.1	7.7	7.2	9.0
White blood cells (*n* ×10^3^/ul)	35.8	32.7	6.2	12.57
Neutrophils (*n*/ul)	32,578	28,449	4,340	9,050
Bands (*n*/ul)	-	654	2	-
Monocytes (*n*/ul)	1,432	1,308	682	377.1
Lymphocytes (*n*/ul)	1,432	2,289	1,054	3142.5
Platelets (*n*/ul)	403,000	303,000	791,000	192,000
Alanine transaminase (iu/l)	49	17	7	16
Alkaline phosphatase (iu/l)	122	161	124	72
Globulins (g/dl)	4.1	4.7	4.0	4.7
Creatine kinase (iu/l)	106	155	122	766
Hepatozoon spp. PCR (copies/ml)	180	2,280	^*^	4,430
Neospora caninum IFA	Positive (1:400)	-	Negative^**^	-
Toxoplasma IgG, IgM titers	Negative	-	(Positive–IgG 1:16)^**^	-

* *Hepatozoon pcr was not performed. Dog 3 was diagnosed via microscopic visualization of intracellular hepatozoon gamont on blood smear.*

** *Patient was tested 1 year post-diagnosis of ach in response to development of seizures.*

*pcr, polymerase chain reaction; ifa, immunofluorescent assay*.

On presentation, hyperesthesia of the sternum, neck, and thoracolumbar vertebral column was demonstrated with palpation. Bilateral grade I/IV medial patellar luxations were also noted. Clinicopathologic findings were similar to those previously described, characterized by a severe mature neutrophilia (32,578/uL [3,000–11,500]), mild monocytosis (1,432/uL [150–1,250]), and normocytic normochromic non-regenerative anemia (31%). *Toxoplasma gondii* IgG and IgM titers were negative and *Neospora caninum* immunofluorescence assay titers were positive (1:400). The positive titer result for *Neospora caninum* was not reported until after the diagnosis ACH had been made by PCR (see below). This was assumed to be clinically insignificant (13, 14).

**Figure 1:**
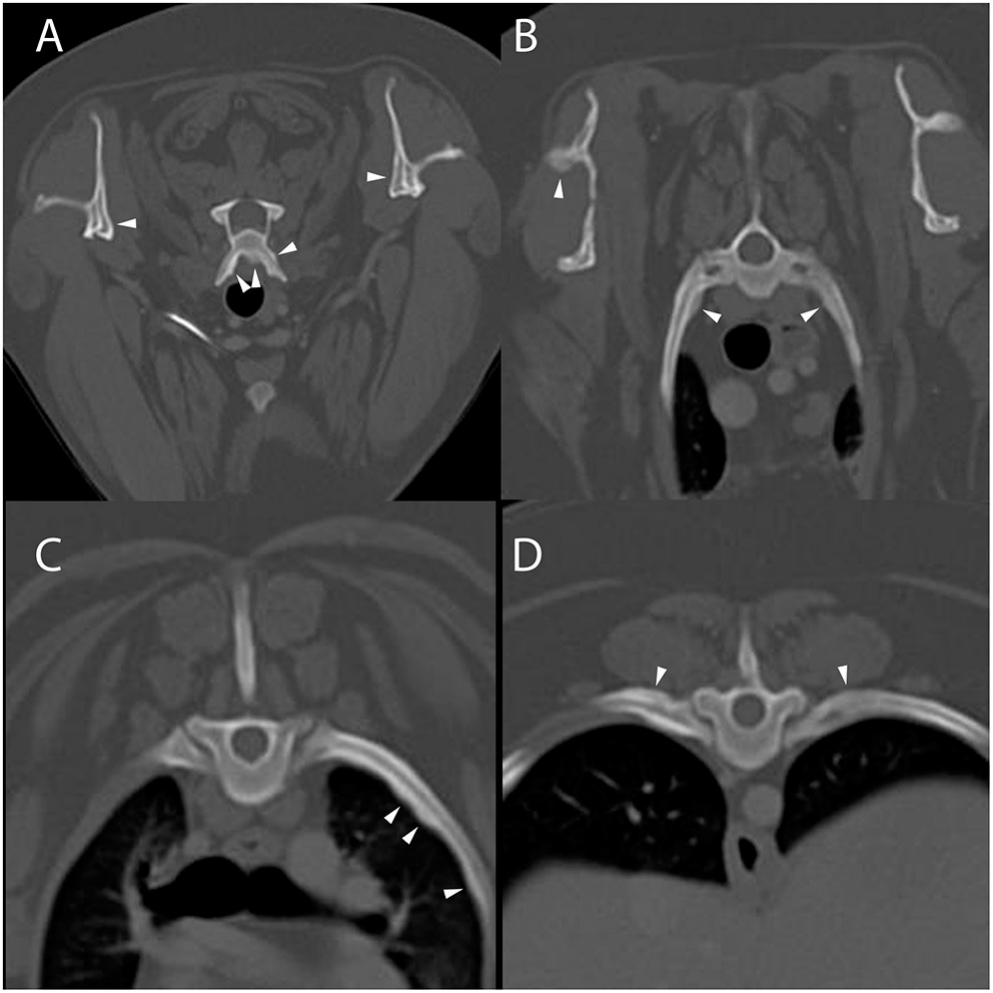
Example of suspected lesions of American canine hepatozoonosis. Post-contrast transverse images at the level of the sixth cervical **(A)**, second thoracic **(B)**, fifth thoracic **(C)**, and tenth thoracic vertebrae **(D)** in a in a 2 year-old female spayed miniature Australian shepherd dog who tested positive for *Hepatozoon americanum* polymerase chain reaction. In all images, there is multifocal, irregularly marginated, periosteal proliferation on several non-articular surfaces of the axial skeleton (white arrowheads). There is no lysis of cortical bone in any image. In **(A)**, the ventral vertebral body and transverse processes are asymmetrically affected. The medial aspect of both scapulae have smooth periosteal proliferation that has organized to form a pseudocortex. In **(B)**, the periosteal proliferation of the spine of the right scapula appears similar to the lesions on the ribs and vertebrae. The second pair of ribs have mild lesions. In **(C)**, the left fifth rib has multifocal regions of irregular periosteal proliferation on the medial surface, while in **(D)** there is periosteal proliferation of the dorsal surface of the tenth ribs. There is no contrast enhancement seen in the soft tissues surrounding the periosteal proliferations.

Computed tomography of the cervicothoracic vertebrae was performed (Figure 1). The dog had multifocal, bilaterally asymmetric, irregularly marginated, non-destructive, non-articular, periosteal proliferative lesions involving the cervical and lumbar vertebrae, scapulae, ribs, and ilia. There was pseudocortex formation involving the subscapular fossae. Additional findings included spondylosis deformans, mineralization of the dorsal longitudinal ligament at the seventh cervical and first thoracic vertebrae, mild non-compressive protrusion of the L6–L7 and L7–S1 intervertebral discs, and a small defect in the infraspinatus fossa of the right scapula (of unknown etiology, Figure 1). The spondylosis deformans was differentiated from the ACH-associated periosteal proliferation because of its distant location from the vertebral endplates. Non-osseous CT findings included subtle soft tissue attenuating foci in the peripheral lungs (of unknown etiology) and multifocal lymphadenomegaly involving the medial iliac, internal iliac, sacral, axillary, left accessory axillary, sternal, and cranial mediastinal lymph nodes. An ultrasound-guided aspirate of an enlarged axillary lymph node was performed, and cytology was consistent with reactive lymphoid hyperplasia. Following CT, PCR (Auburn University College of Veterinary Medicine, Pathobiology Diagnostic Services) was positive for *H. americanum* with 180 copies/mL of blood.

The dog was discharged with gabapentin (10 mg/kg by mouth every 8 h as needed for pain) and prednisone (0.5 mg/kg/day by mouth for 2 weeks then tapered over 2 more weeks). Triple therapy (TMS 15 mg/kg by mouth every 12 h, pyrimethamine 0.25 mg/kg by mouth every 24 h, and clindamycin 10 mg/kg by mouth every 8 h) was prescribed for 2 weeks and the primary care veterinarian was instructed to prescribe decoquinate (10–20 mg/kg by mouth every 12 h) for at least 2 years. The dog's health status was reported to be static at the time of decoquinate initiation 2 weeks later and was subsequently lost to follow-up.

### Dog 2

A 2-year-old male castrated Rhodesian ridgeback weighing 52.7 kg presented for a 4-week history of neck and back pain. The dog lived in northeastern Oklahoma. Five months prior to presentation, an MRI and surgical removal of a dermoid cyst at the level of the third cervical vertebrae was performed. The dog was managed postoperatively for a surgical wound infection with multi-drug resistant *Staphylococcus aureus*.

On presentation, the dog was pyrexic with mild pain appreciated with cervical flexion. An orthopedic exam was unremarkable. A vector-borne disease panel was reported to be negative. Serial complete blood count revealed a moderate neutrophilia (28,449/uL [2,060–10,600]) with a left shift (654 bands/uL [0–300]) and a mild monocytosis (1,308/uL [0–840]). Serum chemistry revealed mildly elevated alkaline phosphatase (161 IU/L [5–131]), hyperglobulinemia (4.7 g/dL [1.6–3.6]) and low creatinine phosphokinase (52 IU/L [59–895]). Urinalysis, thoracic radiographs, and abdominal ultrasound were unremarkable. The dog was hospitalized and treated with intravenous ampicillin sodium/sulbactam. After 3 days, the dog was discharged with enrofloxacin (10.3 mg/kg by mouth every 24 h for 10 days) and amoxicillin/clavulanate (14.2 mg/kg by mouth every 12 h for 10 days).

On recheck, 10 days later, the dog was febrile, but no hyperesthesia was noted. The dog had also developed secondary anterior uveitis, with aqueous flare and severe iritis noted bilaterally. Repeat complete blood count and serum chemistry were similar to initial presentation with a leukocytosis characterized by moderate neutrophilia (22,880/uL [2,060–10,600/uL]), monocytosis (1,716/uL [0–840/uL]), and hyperglobulinemia (4.1 g/dL [1.6–3.6 g/dL]). Creatinine phosphokinase was within normal limits (155 IU/L [59–895]). Urinalysis and cultures of urine and blood had no evidence of bacterial growth.

**Figure 2:**
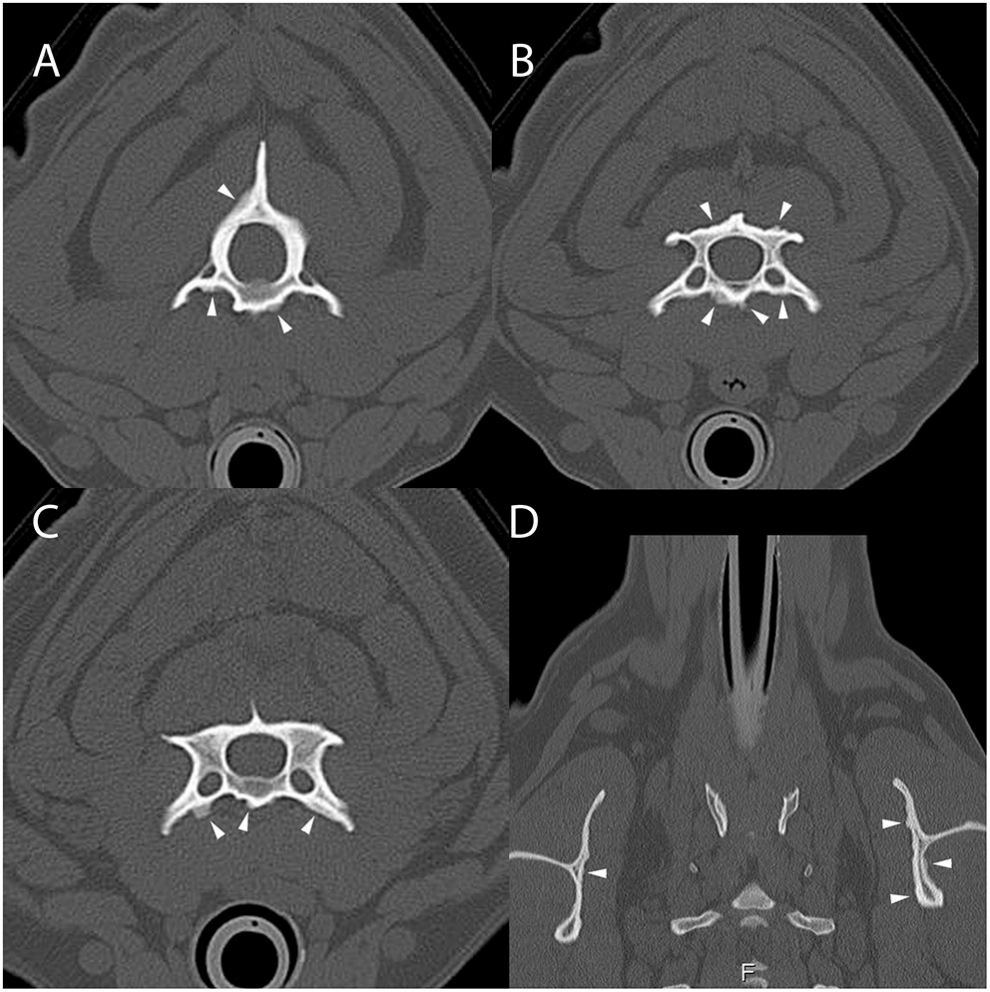
Example of suspected lesions of American canine hepatozoonosis. Non-contrast transverse images of the second **(A)**, third **(B)**, and fourth cervical vertebrae **(C)** and a dorsal image at the level of the scapulae **(D)** in a 2 year-old male castrated Rhodesian ridgeback dog that tested positive for *Hepatozoon americanum* polymerase chain reaction. In all images, there is multifocal, irregularly marginated, periosteal proliferation on several non-articular surfaces of the vertebrae (white arrowheads). In **(A)**, the ventral vertebral body, transverse processes and dorsal arch are asymmetrically affected. In **(B)**, the lamina and ventral vertebral body have similar lesions that vary in thickness depending of the location. In **(C)**, the periosteal proliferation is restricted to the ventral surface of the vertebra. None of the lesions bridge the space between adjacent vertebrae. In **(D)**, the scapulae have mild asymmetric lesions on the medial aspect. The left scapula also has periosteal proliferation of the infraspinous fossa.

Computed tomography of the cervicothoracic vertebrae was performed (Figure 2). The dog had periosteal proliferative lesions that were morphologically similar to those seen in dog 1 involving the cervical vertebrae, second thoracic vertebra, and scapulae, as well as mild cranial mediastinal lymphadenomegaly. The previous surgical site had mildly heterogeneous attenuation and contrast enhancement, which was thought to most likely represent normal post-surgical healing. Following CT evaluation, PCR (Auburn University College of Veterinary Medicine, Pathobiology Diagnostic Services) was positive for *H. americanum* with 2,280 copies/mL of blood. The dog was prescribed 2 weeks of triple therapy (TMS 15 mg/kg by mouth every 12 h, clindamycin 10 mg/kg by mouth every 12 h, and pyrimethamine 0.25 mg/kg by mouth every 24 h) and the primary care veterinarian was instructed to prescribe long-term decoquinate (10–20 mg/kg by mouth every 12 h for 2 years), as well as atropine sulfate 1% (1/4 inch strip applied to each eye every 12 h for 2 weeks) and neomycin/polymixin B/dexamethasone (1 drop in each eye every 6 h for 2 weeks) for management of anterior uveitis. At the time of writing this manuscript, the dog is reported to be markedly improved with resolution of clinical signs on maintenance therapy.

### Dog 3

A 5-year-old, intact female, miniature Schnauzer weighing 6 kg presented for a 3-month history of intermittent lethargy, inappetence, and stiffness when walking. The dog lived in south/central Texas. Episodes of illness would temporarily improve following administration of corticosteroids and various undisclosed antibiotics. On physical examination, the dog was depressed. Tapeworm segments (*Dipylidium caninum*) were also identified in the perianal region. Complete blood count, serum chemistry, and urinalysis revealed a mild hyperglobulinemia (4.0 g/dl), thrombocytosis (791,000/uL [200,000–500,00]) and proteinuria (100 mg/dL). Creatine phosphokinase was normal (122 IU/L [68–400]). A neurologic exam localized a left-lateralized lesion in the C1–C5 spinal cord segment. Cervical hyperesthesia and proprioceptive deficits were noted in the left forelimb and the hind limbs bilaterally. Abdominal ultrasonography revealed no significant findings. The dog was discharged with instructions for strict crate rest.

One week later, the dog presented for a recheck. A repeat neurologic exam revealed decreased nasal sensation and quadrilateral conscious proprioceptive deficits. Cerebrospinal fluid analysis yielded no significant findings. On a peripheral blood smear, a single intracellular Hepatozoon gamont was identified. Cervical and thoracolumbar spinal radiographs revealed no significant findings.

**Figure 3:**
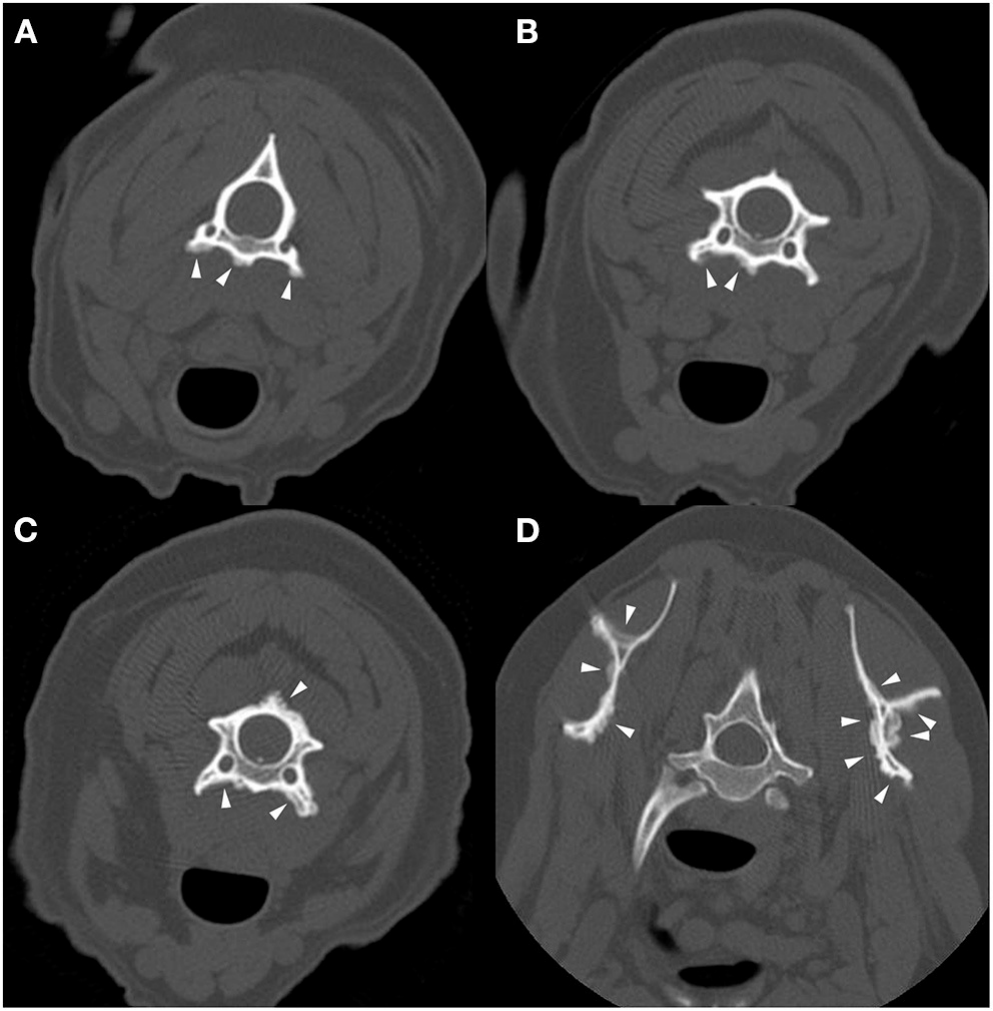
Example of suspected lesions of American canine hepatozoonosis. Non-contrast transverse images of the second **(A)**, third **(B)**, and fourth cervical vertebrae **(C)**, as well as the first thoracic vertebra and scapulae **(D)** of a 6 year-old intact female miniature schnauzer dog in which a *Hepatozoon americanum* gamont was identified on a peripheral blood smear. In all images, there is multifocal, irregularly marginated periosteal proliferation on several non-articular surfaces of the vertebrae (white arrowheads). The underlying cortical margins are smooth, intact, and of normal thickness. In **(A)**, the ventral vertebral body and transverse processes bilaterally have similar lesions that do not connect. In **(B)**, the periosteal proliferation is lateralized to the right ventral vertebral body and transverse process. In **(C)**, similar lesions are also seen on the dorsal spinous process and lamina. In **(D)**, both medial and lateral surfaces of both scapulae are affected. This is an example of the marked asymmetry and irregularity of the periosteal proliferation.

Computed tomography of the cervical vertebrae and head was performed (Figure 3). The dog had lesions that were morphologically similar to those seen in dogs 1 and 2 involving the cervical vertebrae, first thoracic vertebra, and scapulae. There was also mild non-compressive protrusion of the C6–7 intervertebral disc. The dog was discharged with triple therapy (TMS 12 mg/kg by mouth every 12 h, clindamycin 9.4 mg/kg by mouth every 8 h, and pyrimethamine 0.18 mg/kg by mouth every 24 h), meloxicam (0.14 mg/kg by mouth once followed by 0.075 mg/kg by mouth every 24 h) and long-term decoquinate (20 mg/kg by mouth every 12 h indefinitely). The dog has since been seen for numerous follow-up appointments due to the development of idiopathic epilepsy, bone marrow suppression from sulfa drug administration, immune-mediated neutropenia, and several clinical relapses of ACH. At the time of writing this manuscript, the dog's clinical signs are well-managed 6 years since initial diagnosis with daily decoquinate and ponazuril (8.3 mg/kg by mouth every 12 h for 2 weeks) prophylactically around stressful events or medical procedures.

### Dog 4

A 7-year-old, male neutered, Yorkshire terrier mix weighing 4 kg presented for chronic non-specific hyperesthesia, lethargy, and fever. The dog lived in northern Alabama. The dog was previously a stray and displayed clinical signs for the full duration of ownership, which was ~10 months. At presentation, he was receiving carprofen, doxycycline, and clindamycin with minimal improvement. On physical exam, the dog was tense on abdominal palpation and had lumbosacral pain. A complete blood count was unremarkable. The dog's neutrophil count was normal (9,050/ul [3,100–11,800]). Serum chemistry and urinalysis were unremarkable. A point-of-care tick-borne pathogen and heartworm test (SNAP 4Dx; IDEXX Laboratories, Inc. USA) was negative for all organisms. Brucella immunofluorescence assay and agar gel immunodiffusion were both negative. Thoracic radiographs revealed a mild diffuse bronchial pattern. Abdominal radiographs identified mild, multifocal, smoothly marginated, periosteal proliferation along both femurs, which was most apparent proximally. An abdominal ultrasound examination noted gallbladder debris, mild left renal pyelectasia, and mild medial iliac and jejunal lymphadenopathy. Fine-needle aspirates of the left medial iliac lymph node, prostate, and spleen revealed no signs of relevant disease.

**Figure 4:**
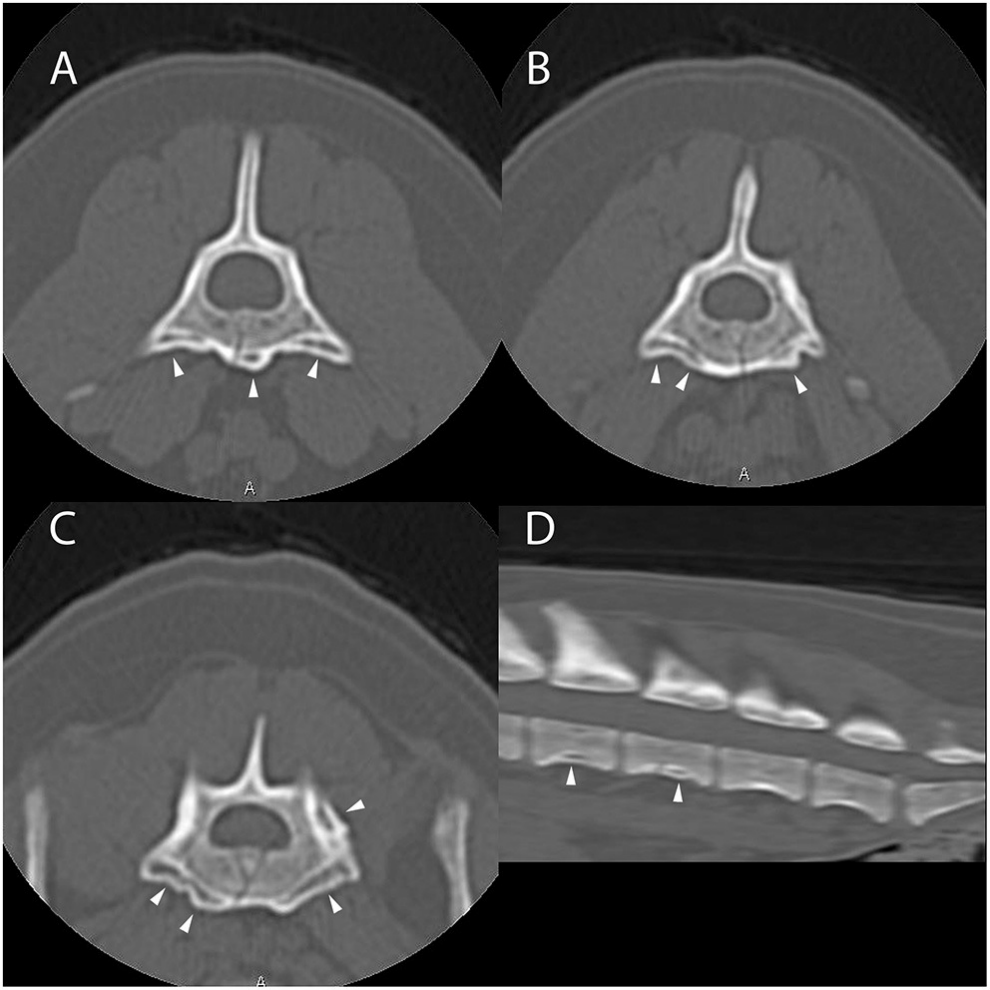
Example of suspected lesions of American canine hepatozoonosis. Non-contrast transverse images of the fifth **(A)**, sixth **(B)**, and seventh lumbar vertebrae **(C)** and a sagittal image of the caudal lumbar vertebrae **(D)** in a 7 year-old male neutered Yorkshire terrier dog that tested positive for *Hepatozoon americanum* polymerase chain reaction. In all images, there is multifocal, irregularly marginated, periosteal proliferation on several non-articular surfaces of the lumbar vertebrae (white arrowheads). There is no lysis of cortical bone in any image. In **(A)**, the lesions are restricted to the ventral vertebral body and transverse process. The periosteal proliferation is thick and forms a nearly continuous pseudocortex. The lesions in **(B)** are similar to **(A)**, but highlight the asymmetry of the lesions. In **(C)**, the pseudocortex is undulant and disappears axially. The periosteal proliferation is lateralized and equally prominent on the left pedicle of the sixth lumbar vertebra. In **(D)**, smooth periosteal proliferation is present at the midventral vertebral body of the fourth and fifth lumbar vertebrae. The periosteal proliferation is widest at the middle of the vertebral body and tapers toward the intervertebral disc space. There is no bridging periosteal proliferation.

Computed tomography of the thoracic, lumbar, and sacral vertebrae was performed (Figure 4). The dog had multifocal, asymmetric, periosteal proliferative lesions with pseudocortex formation involving the lumbar vertebrae. The dog was discharged on carprofen.

On recheck (107 days after initial presentation), the dog was pyrexic with a similar physical exam. Plasma protein was elevated (9.0 g/dL [6.0–7.5]). Serum chemistry revealed hyperglobulinemia (4.7 g/dL [2.1–4.3]) and an elevated creatine phosphokinase (766 U/L [50–300]). Serological C-reactive protein was elevated (>60 mg/dL [<9.9]). Arthrocentesis and cytology of carpi and stifles was unremarkable. Bronchoalveolar lavage cytology was consistent with mucinous hyperplasia, negative for Mycoplasma PCR, and did not culture organisms. Cerebrospinal fluid analysis was unremarkable. Renal pelvis cytology was unremarkable and the culture result was negative. Polymerase chain reaction (Auburn University College of Veterinary Medicine, Pathobiology Diagnostic Services) was positive for *H. americanum* with 4,430 copies/mL of blood. The dog was prescribed 2 weeks of triple therapy (TMS 15 mg/kg by mouth every 12 h, clindamycin 10 mg/kg by mouth every 8 h, and pyrimethamine 0.25 mg/kg by mouth every 24 h) followed by 2 years of decoquinate (15 mg/kg by mouth every 12 h). Three months after initial diagnosis, the dog was reported to be doing well and without clinical relapse. The dog has since been lost to follow up at the time of submitting this manuscript.

## Discussion

All 4 dogs with ACH reported herein had multifocal, bilaterally asymmetric, irregularly marginated, non-destructive, non-articular, periosteal proliferative lesions involving the vertebrae. Osseous lesions caused by *Hepatozoon americanum* are described grossly and histologically as irregular, circumferentially arranged plaques of spongey bone deposited around intact cortical bone (7). The CT changes in all 4 cases are consistent with this finding, including pseudocortex formation, which is thought to be an indication of chronicity (Figures 1, 4) (7).

While the axial skeleton is reported to be less commonly affected than the appendicular skeleton (7), all 4 cases had multiple lesions affecting the vertebrae. A previous study of gross necropsy found that axial lesions were most often seen on the lateral surfaces of the vertebral arches and dorsal spinous processes (7). Our cases contrast this, as the majority of vertebral lesions identified were on the transverse processes and ventral vertebral bodies (Figures 1, 3, 4). Some cases in this report only had osseous lesions detected via CT, even when prior radiographs included the same region in the field of view. Due to the distribution of lesions on the transverse and spinous processes, it is possible that the lesions were not detected because of summation.

Osseous lesions of ACH could be misinterpreted as spondylosis deformans, a degenerative disease of the vertebral endplate associated with periosteal proliferation that attempts to bridge the disco-vertebral junction (15). It is a common finding with up to 75% noted to be affected at necropsy (15). Spondylosis deformans (SD) is typically considered clinically insignificant unless associated with intervertebral disc herniation or spinal nerve impingement (15, 16). In contrast to ACH, proliferations associated with SD are centered on the intervertebral disc space and are limited to the vertebral column. It is possible that both lesion distributions can be present in a dog with ACH and satisfaction of search bias may cause the ACH lesions to be overlooked. Other generally inconsequential potential interpretations of ACH lesions include osteophytosis associated with osteoarthritis and chronic fracture healing (especially rib fractures) (7). Due to relatively low prevalence and restricted geographic range, there is the potential that ACH-associated periosteal changes may be misinterpreted or deemed clinically irrelevant.

Hypertrophic osteopathy (HO) is another important differential diagnosis for ACH (17). Both diseases cause periosteal proliferation affecting long bone diaphyses. When examined histologically, the osseous changes associated with the two are nearly identical, including pseudocortex formation (7). Hypertrophic osteopathy is usually triggered by an intrathoracic lesion, but the exact mechanism is unknown. While HO often starts distally, with lesions first developing in the metacarpals and metatarsals, these bones are rarely affected in ACH (7). The presence of vertebral and rib lesions may be used clinically to differentiate ACH from HO and determine if further imaging to identify a primary thoracic or abdominal mass is indicated.

Other causes of periosteal proliferation could potentially be confused with ACH. For example, stress fractures in racing greyhounds may be associated with bilateral periosteal proliferation of the acetabula or long bones (18, 19). Stress fracture can be differentiated from ACH by the presence of a fracture line, location of lesions, and absence of systemic signs. Another cause of periosteal proliferation on CT is subperiosteal hematoma (20). Subperiosteal hematomas can be differentiated from ACH by their smooth margins, mineralized outer rim, location, and association with trauma or coagulopathy. Hematogenous bacterial osteomyelitis is another uncommon cause of periosteal proliferation in dogs (21). Clinical and CT findings may overlap with ACH, but cortical lysis and medullary bone lysis and sclerosis are often seen in osteomyelitis and were not seen in these dogs with ACH. Finally, metastatic neoplasia is another important cause of multifocal periosteal proliferation in dogs (22). Metastatic lesions would also be expected to have cortical lysis (22). Periosteal proliferation, in general, is caused by stimulation of periosteum to produce new bone by infection, trauma, neoplasia or systemic disease (23). The type of periosteal proliferation depends on the nature of the inciting cause and rate of new bone formation (23). The underlying mechanism for the periosteal proliferation of ACH is currently unknown. One proposed theory implicates local myositis and periostitis associated with organisms encysted in adjacent muscle, but histologic examination has consistently failed to identify any organisms associated with osseous lesions (7, 24). None of the cases reported here demonstrated contrast enhancement in skeletal muscle adjacent to the periosteal lesions, as would be expected with a local myositis. An alternative theory factors in an unusual trait in the *H. americanum* life cycle, in which the infection and transformation of a “host-cell” causes aberrant production of mucopolysaccharides that produce the classic “onion skin” cysts in striated muscle. It is hypothesized that these cells also release growth factors and inflammatory mediators into systemic circulation that promote periosteal proliferation (7). This theory may be supported by the disseminated bilateral distribution and abrupt onset of ACH lesions, as well as their similarities to the lesions seen in HO.

As CT becomes more accessible in veterinary medicine, it is proving to be a useful imaging modality in cases of systemic febrile illness and generalized non-specific musculoskeletal pain. There are many differential diagnoses for such a clinical presentation, and due to this, a recognizable pattern of osseous changes will help obtain an earlier diagnosis, expedite treatment, and reduce extraneous diagnostic testing. A limitation of our report is the lack of appendicular skeleton imaging, as this disease is historically diagnosed using the presence of ACH-related lesions on long bones.

There are several strengths of this manuscript. First, the multicenter approach allowed us to include more dogs and incorporate diverse perspectives in interpreting the results. Second, the means of diagnosis (by PCR detection of pathogen DNA and cytology) are highly specific and not highly sensitive (9). This means that it is highly likely that all 4 dogs in this report actually had ACH. Furthermore, in clinical practice, CT could aid in making the diagnosis of ACH even when PCR and cytology fail. Therefore, it is crucial that image evaluators are aware of CT changes in dogs with ACH.

There are a few important limitations of this manuscript. Despite including multiple centers, the number of cases is low. This is thought to be caused by the relatively low prevalence of ACH and the fact that the diagnosis often does not require CT. The cases were recruited from 3 different institutions over >10 years. Because of the retrospective nature of the study, treatment protocols were not standardized. For example, in some cases, decoquinate was prescribed for 2 years, while in others it was prescribed indefinitely. That being said, the prescribing veterinarians generally followed the guidelines of the Companion Animal Parasite Council regarding drug selection and dosage (25). Furthermore, this weakness is not relevant to the primary aim of this manuscript, which is to describe CT findings in dogs with ACH.

In conclusion, ACH should be considered in dogs with fever, muscle wasting, myalgia, and neutrophilia. Expected CT findings include multifocal, bilaterally asymmetric, irregularly marginated, non-destructive, non-articular, periosteal proliferative lesions involving the vertebrae, ribs, scapulae, and ilia. Specific testing for *H. americanum* (PCR, buffy coat analysis, and/or skeletal muscle biopsy) is recommended in any dog with this constellation of clinical signs and CT findings.

## Data Availability Statement

The original contributions presented in the study are included in the article/Supplementary Material, further inquiries can be directed to the corresponding author/s.

## Ethics Statement

Ethical review and approval was not required for the animal study because it was a retrospective study. The owners of the animals consented to allow the use of their medical records and archived diagnostic images. Written informed consent was obtained from the owners for the participation of their animals in this study.

## Author Contributions

JG, CC, JE, and JL contributed to the conception and design of the study. JG and CC created case criteria. JG, CC, JE, AL, and DD identified appropriate cases for case descriptions. CC wrote the first draft of the manuscript. JE wrote sections of the manuscript. JG created figures. CC and JG wrote figure descriptions. All authors contributed to manuscript revision, read, and approved the submitted version.

## Conflict of Interest

The authors declare that the research was conducted in the absence of any commercial or financial relationships that could be construed as a potential conflict of interest.

## Publisher's Note

All claims expressed in this article are solely those of the authors and do not necessarily represent those of their affiliated organizations, or those of the publisher, the editors and the reviewers. Any product that may be evaluated in this article, or claim that may be made by its manufacturer, is not guaranteed or endorsed by the publisher.
