# Supplementary material for: American Canine Hepatozoonosis Causes Multifocal Periosteal Proliferation on CT: A Case Report of 4 Dogs
Source: Front Vet Sci. 2022 Apr 27;9:872778. doi: 10.3389/fvets.2022.872778 (PMC9093736; doi:10.3389/fvets.2022.872778)
Supplement: Supplementary file 1 [file Data_Sheet_1.pdf]

Figure S1. Timeline of diagnostic assessments and time to intervention for four dogs with American canine hepatozoonosis.

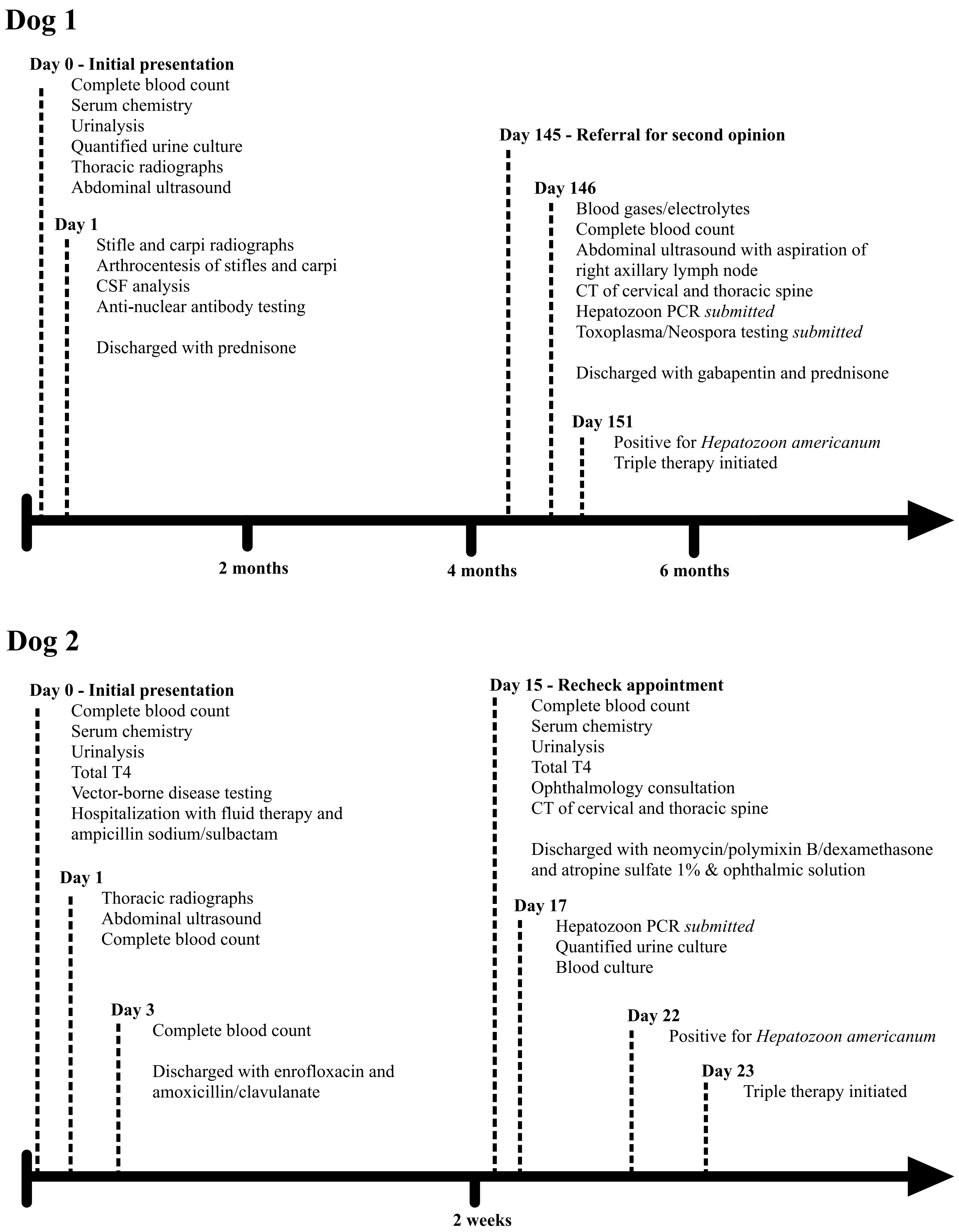

Dog 3

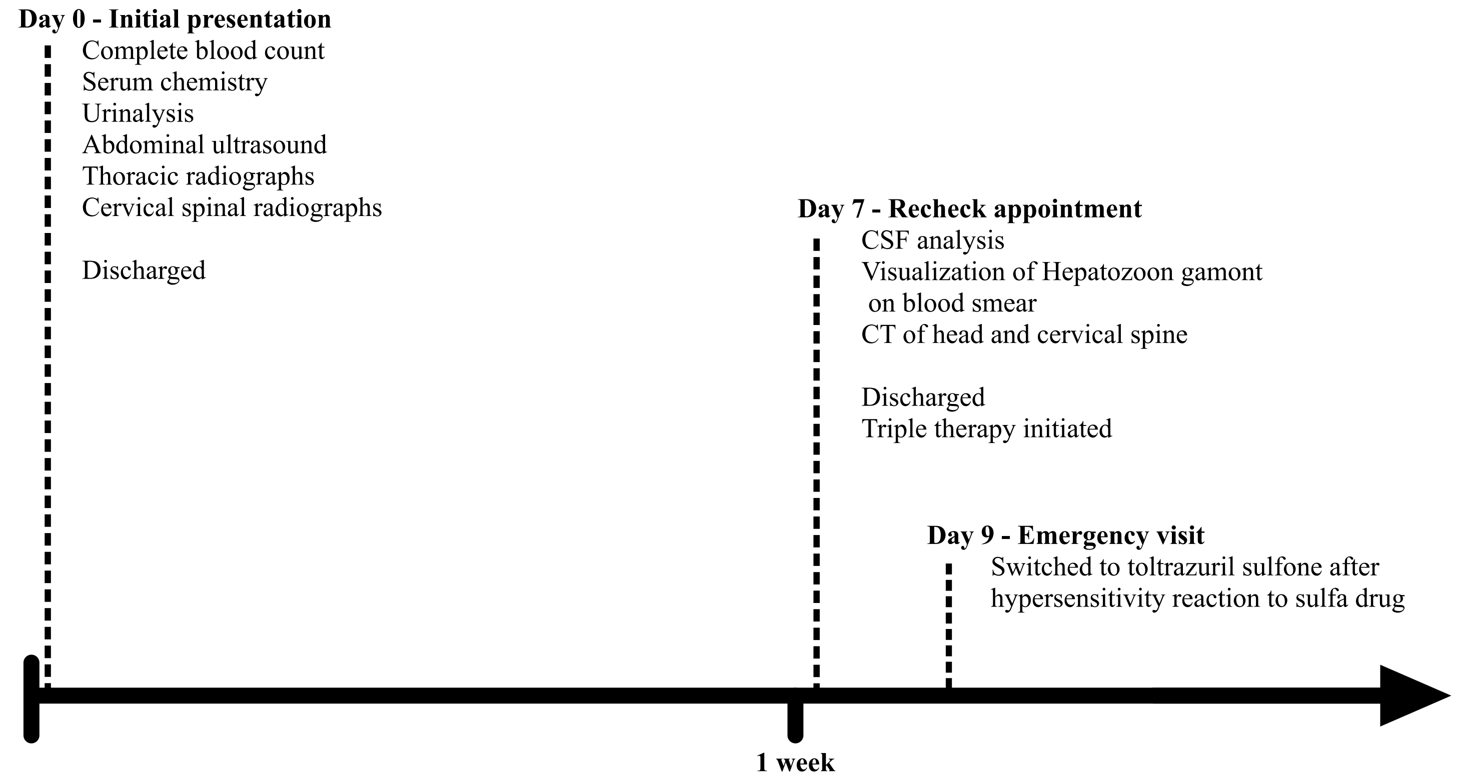

Dog 4

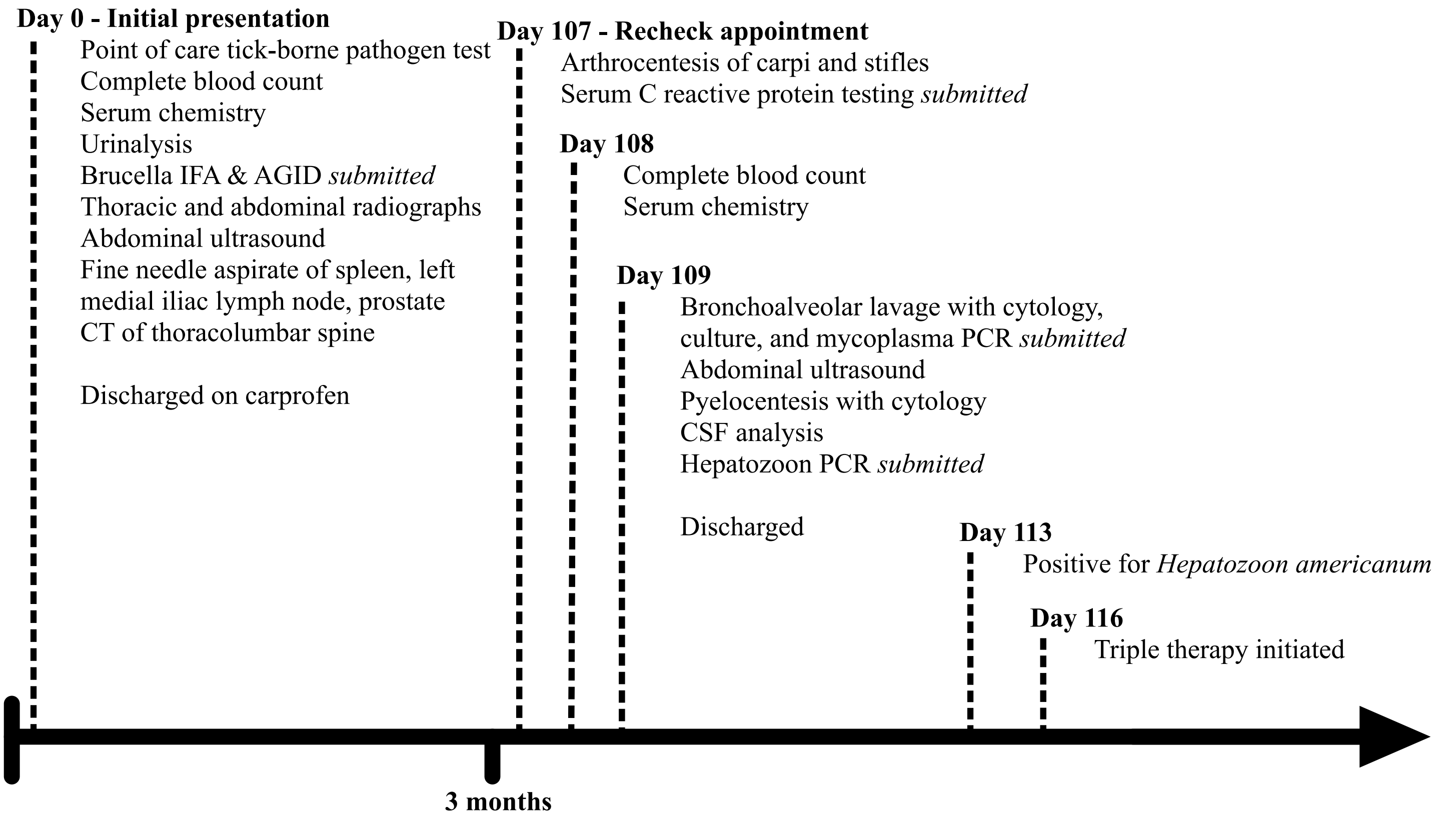

Abbreviations: CSF - cerebrospinal fluid, CT - computed tomography, PCR - polymerase chain reaction, IFA - immunofluorescent assay, AGID - agar gel immunodiffusion
